# Tuberculosis

**Published:** 1909-01

**Authors:** George Brown

**Affiliations:** Atlanta, Ga.


### TUBERCULOSIS.*

BY GEORGE BROWN, M. D., ATLANTA, GA.

Tuberculosis. A name I never mention without a shudder, and think with Dr. Huber that: "It is with a real sense of melancholy that one contemplates the long death-roll of those of the world's great men and women who have succumbed untimely to the tubercle bacillus, which is and has been through countless generations by far the most potent of all death-dealing agencies. Had it not been for this detestable parasite Bastien Le Page might have given us another Joan-of-Arc to feast our eyes upon; Rachel might for many years have continued to permeate the spirits of her audiences with the divine fire that was in her. Our navy did well enough in the 1812 war, as all the world knows;

*Read before Southern Medical Association, Atlanta, Ga.

but what a rip-roaring time there would have been if John Jones had lived to take a hand in it. We might be reading some more of Robert Crane's splendid war stories; we might have had some of Robert Louis Stevenson's delicious lace-work; Schiller might have given us another Song of the Bells; we might have taken another "Sentimental Journal," with Laurence Sterne; Henry Cuyler Bunner might have continued to delight us, and to touch our hearts; John Keats might have given us another "Endymion." Had the tubercle bacillus permitted, Nevin might have vouchsafed us another "Rosary;" Von Weber another "Euryanthe Overture;" Chopin might have dreamed another "First Polonaise;" and the tender flute notes of Sidney Lanier might even now be heard. Maria Constantinovna Bashirtseff, Lavier Bischat, John Godman, Rene Theophile Hyacinth Laennac, Henry Purcell, John Sterling, Henry Timrod, Artemus Ward, Henry Kirks White, Henry David Thoreau, Baruch Spinoza—such names as these are but a moity among those of the world's nobility, whose precious lives were cut off in their prime by the "Great White Plague."

When a student looks back for the past nine years and reflects on what the world has done to alleviate the tubercular unfortunates, and realizes what the future has in store for them, we can but offer thanks for those devoted men and women who have been the pioneers in this fight, and can but pause a moment to give praise to those to whom praise is due.

I saw the birth of the first organized association in the United States to fire the opening guns of the campaign, which reverbated from Maine to Mexico. Since that time having put my hand to the plough I have never looked back.

Five years ago I started the agitation for a State Sanitarium for consumptives in this State, with little assistance from those who could give it and personal and general abuse from others. I have worked on until I can see an assurance that it will yet be built.

In submitting my name as a candidate for the House of Representative from Fulton County last June, I was overwhelmingly elected, heading the ticket with eleven opponents in the field by over 500 votes. This without any solicitation of votes on my part, only stating that I made the race *purely in the interest of the tubercular poor.*

The educational campaign can be said well established. There is no need, however, of any politician and "wise acre" jumping in and rushing into the front to educate the dear people.

What the dear people want is not education, but bread and meat and a place to sleep and medical attention for the tubercular poor. It is absolutely horrifying to read the Sunday papers and Magazines of today and see what harm they are doing. They are preaching to these poor unfortunates that fresh air and such rot is all any one needs to cure this disease.

In the name of God where are the people they have cured—listen to plain statistics. Averages cures incipient cases given proper Sanitarium treatment 60 to 74 per cent. Average cures home treatment, 1 per cent. Yet these miserable would be reformers are daily preaching to these people that there is nothing else that they need.

I beg to refer to the work of a plain elegant Atlanta gentleman and physician, Dr. Louis Rouglin, who helped to organize the first State Society here to fight this disease whose experience with this disease is not on paper, but who has turned out a large per cent. of cures.

He assisted in founding the first institution on modern lines in the South, The Pine Ridge Sanitarium, and it will stand near this city as a monument to his work and skill. These are the men who should have the proper credit for their work. Men who are curing patients and saving lives not writing newspaper stories.

I wish to cite your attention to the following cases, which I will be glad to show any one interested in this work.

Case 1. A. C. B., male, age 30, merchant, one brother died of tuberculosis in 1888, and in 1906, otherwise family history negative, past history negative.

Present History.—Contracted a cold latter part of March, 1908, coughed a good deal, took patent medicine for cough, but received no relief, began losing flesh rapidly and spit up blood on several occasions, had night sweats, and lost appetite, was growing weak, is troubled with shortness of breath, cough became worse and troublesome, patient lost 14 pounds, night sweats became exhausting and patient consulted a physician and he was told he had tuberculosis.

On Admission.—Patient looked emaciated, weak and short of breath, says he feels exhausted, cannot sleep on account of cough and night sweats, complains of pain in chest, and expectorates a yellowish tenacious sputum of a foul odor, slightly blood tinged, tongue coated, weight of patient 121 pounds. Temperature at 4:30 P. M., 103 degrees, pulse 120, respiration 28.

Physical Examination.—Several signs of marked consolidation at upper right lobe, and evidence of cavity formation and evidence of involvement of lower right and upper left in a lesser degree; examination of sputum reveals numerous tubercle bacillis; opthalmo-tuberculin test positive.

Subsequent History.—Patient was admitted July 10th, 1908 and continued to have marked evening elevation of temperature varying from 99.4 to 102.6 degrees F. until the 20th of July, when temperature became normal and continued so with the exception of a slight disturbance, this was soon corrected and temperature remained normal to date of patients discharge from the Sanitarium. Cough became less troublesome, on the 6th day after admission and finally ceased all together, night sweats stopped one week after admission, appetite improved and physical signs began to disappear, repeated examination of sputum since October 20th, failed to reveal any tubercle bacilli, and patient was dismissed November 8th, weighing 145 1-2 pounds, a gain in weight of over 24 pounds. Length of staying in Sanitarium, four months.

Case 4. T. H., femade, age 14, school girl. Family and previous history negative. Present history began coughing and losing flesh a year ago, and troubled with night sweats, lost about 12 lbs. in the last ten months, had a hemorrhage and consulted a physician.

On admission to the Sanitarium, patient looked thin, markedly anaemic and nervous, complains of pain in chest and under shoulder blade and is troubled with shortness of breath, and hoarseness, physical examination reveals involvement of both upper and lower left lobe, examination of sputum revealed T. B., opthalmo-tuberculin, test negative with 1-2 of 1 per cent. solution, positive later with 1 per cent. solution, evening temperature on July 19th, date of admission 100.20 F. Pulse 96 R. 24.

Subsequent History.—Patient continued to show evening elevation of temperature varying from 99 to 100.20 F. to August 22, when it became and continued normal to September 15th, when it again showed an evening rise to the 21st of September, since then patient's temperature was normal to date of discharge from the Sanitarium, cough soon disappeared, and patient had no night sweat during stay at the Sanitarium, anaema improved, and patient gained 30 lbs. in weight, hoarseness and physical signs disappeared and sputum is clear of T. B. Patient was dismissed November 8th. Length of stay, three months and 21 days.

Case 14. W. H. C., male, age 28, Banker. Father died from "Lung Fever" ten years ago, one brother died of tuberculosis two weeks prior to patient's entrance to the Sanitarium. Patient was constantly with his brother during the last three months of his brother's illness. Patient had catarrh and slight cough at last five years, cough has been growing worse, and is troubled with hoarseness, had no night sweats, expectorates in the morning and thick yellowish sputum, no blood, lost weight the last three months, and has an evening rise of temperature.

Physical Examination.—Shows involvement over entire left lung. Opthalmo-tuberculin reaction, and examination of sputum is positive. Patient was admitted on August 19th, run a temperature varying from 99.8 in the morning to 101 and 104 degrees in evening, patient is very weak, eats and sleeps badly, this condition continued with but slight improvement to September 12th. when evening temperature fell to normal, and has remained so, appetite improved and patient rests comfortably, physical signs are rapidly disappearing and patient gained 35 pounds in weight since his admission to the Sanitarium, his last specimen of sputum still contained a small number T. B., patient was advised to remain in the Sanitarium for a short while longer.

In concluding this article, I can say that the facts brought out by the American Anti-Tuberculosis League three years before any other society was formed still stand as truths. That this disease is contagious and curable—that 80 per cent. can be cured if taken in time. That the greatest source of infection in the world is milk and inhaling germs, that climate never cured a living soul. That the Serum treatment offers in the hands of experienced and long trained men, the best results. That it can never be given with safety by a man who has not had a long and careful training in giving it. That the only hope for permanent cure is early diagnosis and sanitarium treatment, usually lasting less than six to twelve months.

That the doctor who does not give the patient a chance for his life by early diagnosis and proper care is a criminal.

